# Skin Interstitial Fluid and Plasma Multiplex Cytokine Analysis Reveals IFN-γ Signatures and Granzyme B as Useful Biomarker for Activity, Severity and Prognosis Assessment in Vitiligo

**DOI:** 10.3389/fimmu.2022.872458

**Published:** 2022-04-07

**Authors:** Chau Yee Ng, Yen-Chuan Chiu, Yu-Pei Chan, Yu-Jr Lin, Pei-Han Chung, Wen-Hung Chung, Cheng-Lung Ku

**Affiliations:** ^1^Vitiligo Clinic and Research Center, Chang Gung Memorial Hospital, Linkou Branch, Taoyuan, Taiwan; ^2^Department of Dermatology, Chang Gung Memorial Hospital, Linkou Branch, Taoyuan, Taiwan; ^3^Graduate Institute of Clinical Medical Science, College of Medicine, Chang Gung University, Taoyuan, Taiwan; ^4^Department of Statistics, Chang Gung University, Taoyuan, Taiwan; ^5^Department of Pharmacology, Elixiron Immunotherapeutics Inc., Taipei City, Taiwan; ^6^Department of Nephrology, Chang Gung Memorial Hospital, Linkou Branch, Taoyuan, Taiwan; ^7^Center for Molecular and Clinical Immunology, Chang Gung University, Taoyuan, Taiwan

**Keywords:** vitiligo, multiplex cytokine assay, biomarker, interferon gamma, granzyme B

## Abstract

**Background:**

The course of vitiligo is unpredictable, with periods of disease flare-ups and prolonged recovery periods. It is essential to establish a biomarker profile as a substitute marker for disease activity to predict disease activity, severity, and prognosis prediction. The use of localized skin interstitial fluid as biomarkers has recently gained interest, but extensive studies of the association between skin interstitial fluid, plasma, and the disease course is lacking. This study aims to evaluate the cytokine expression profiles in the skin and plasma and the utility of the biomarker panel in assessing disease activity, severity, and prognosis in patients with vitiligo.

**Methods:**

In this prospective cohort study, 86 patients and 34 healthy controls were recruited from the outpatient department of a tertiary medical center from March 2019 to September 2021. All patients were of Asian ethnicity. Two independent investigators evaluated disease activity and severity with longitudinal follow-ups for treatment response for a-12 month period. Ultrasensitive multiplex cytokine panel and single-molecule counting technology immunoassays were used to study the cytokine expression in skin interstitial fluid and plasma.

**Results:**

IFN-γ and its’ signature cytokines, including CXCL9, CXCL10, and GzmB, are most highly expressed in the vitiligo patients’ lesion skin interstitial fluid and plasma compared to healthy control. By way of comparison, no significant changes in IL-1β, IL-13, IL-15, IL-17A, IL-18 were observed. Receiver operating characteristic analysis revealed that IFN-γ is the most sensitive and specific marker in predicting disease activity, followed by CXCL10 and GzmB. CXCL-9 was sensitive and specific in diagnosing vitiligo disease severity. The decrease in IFN-γ expression level is positively correlated with the treatment response.

**Conclusion:**

IFN-γ, CXCL9, CXCL10, and GzmB are highly expressed in vitiligo patients’ lesion skin and plasma and may serve as biomarkers for the clinical activity, severity, and prognosis prediction in vitiligo patients. Among all, IFN-γ exerts the highest predictive value in disease activity and treatment response, supporting the critical role of IFN-γ in the pathogenesis of vitiligo.

## Introduction

Vitiligo is a common autoimmune disease that progressively destroys melanocytes in the skin, resulting in patchy disfiguring depigmentation. In recent years, studies have identified several critical pathways contributing to the pathophysiology of vitiligo (1, 2). Studies demonstrated cytotoxic T cells (CTLs) are involved in the melanocyte destruction and the role of IL-15 in memory T cells contributing to the refractory course in vitiligo (3–5). New targeted treatments are developing based on scientific discovery. Compared to other autoimmune skin diseases such as psoriasis and atopic dermatitis, vitiligo lacks prominent inflammatory signs. Objective clinical activity signs such as confetti-like depigmentation, trichome lesions, and Koebner’s phenomenon are subtle and only visible under wood’s lamp examination in lighter skin phototype patients. It is unclear whether these clinical signs can evaluate disease activity over time, and the repigmentation often takes months to years to recover. The disease course of vitiligo is also unpredictable with periods of disease flares. Hence, there is a need for biomarker analysis as a substitute marker for disease activity to predict disease flare-ups and prognosis prediction. Moreover, biomarker analysis of vitiligo patients may also serve as guidance to select patients for targeted treatment.

Recent studies have shown that chemokine CXCL9 CXCL10 is elevated in the plasma of vitiligo patients with active vitiligo and correlated with treatment response (6–10). However, many other cohorts also show an increased cytokine expression of IL-15, IL-17, IL-1β, IL-18, etc. (11–15) The plasma cytokine/chemokine level is a net effect of the whole-body inflammation and can be easily affected by other concurrent autoinflammatory disorders that are present. Directly studying biomarkers from the skin is a better approach. However, conventional sampling using skin biopsy with immunohistochemistry is limited by low infiltrating immune cells, making it difficult to detect and quantify. Strassner et al. first proposed a new minimally-invasive method of skin sampling technique that is non-scarring and easy to perform (6). The method utilizes a negative pressure suction device to create a blister that contains interstitial fluid that resembles local tissue inflammation. The use of localized skin interstitial fluid as biomarkers has recently gained interest, but extensive studies of the association between skin interstitial fluid, plasma, and the disease course is lacking.

In this study, we obtained blisters interstitial fluid from the lesion and non-lesion skin paired with plasma from vitiligo patients and healthy controls to study various cytokine expressions. We further validate these results in a longitudinal cohort with detailed record of clinical phenotype to discover the ideal biomarker for disease course and prognosis prediction.

## Materials and Methods

### Patients Recruitment

A cohort of 86 patients with vitiligo and 34 healthy control individuals were recruited at our department between March 2019 to September 2021. The study was approved by the Ethics Committee of Chang Gung Memorial Hospital (IRB no: 201802265A3).

Inclusion criteria include subjects >20 years old diagnosed with non-segmental, generalized vitiligo who was not on treatment three months before screening. Patients < 20 years old, pregnant, with segmental vitiligo, vitiligo lesions limited to glabrous skin region (ex: mucosal vitiligo, acral tip vitiligo), presence of leukotrichia, and/or presence of other inflammatory skin diseases was excluded from this study. Patients with other systemic autoimmune disorders such as thyroiditis, lupus erythematosus were also excluded. Two independent investigators (CY and YC) diagnosed all patients using wood’s light examination. Healthy volunteers with no history of vitiligo, autoimmune disease, or chronic disorders were included. In all healthy volunteers, we performed a complete body checkup to exclude patients with signs of skin inflammation and laboratory survey on the hemogram, biochemistry, autoimmune panel, thyroid panel before inclusion.

Thirty-three vitiligo patients underwent blister interstitial fluid collection from vitiligo lesion, non-lesion skin, and paired plasma. Twelve healthy controls with paired blister interstitial fluid and plasma were collected for comparison analysis. Plasma was collected from 53 patients with vitiligo and 22 healthy controls for subsequent analysis. A sampling of patient plasma and skin blisters was collected at the first visit to our vitiligo clinic. In 35 patients, serial plasma was obtained at the six months of treatment with a corresponding record of disease improvement using the VES score and photography record. **(** **Supplementary Figure 1****)**. Patients were followed up and evaluated every month for at least 12 months. We recruited sample size of vitiligo patients to healthy volunteers with paired skin blister interstitial fluid and plasma analyte within a 2:1 ratio.

We further classified these patients based on the disease activity, severity, and prognosis. Active vitiligo was defined as the presence of clinical signs of active vitiligo and a Vitiligo Disease Activity score (VIDA) of 4+, indicating the presence of a new spreading lesion within three months. The clinical symptoms of active vitiligo include confetti-like macule, type 2b Koebner, and trichome lesions (16, 17). Stable vitiligo was defined as a VIDA score of 0 to -1, and patients reported no new lesions to arise within a year (18). with the absence of the aforementioned clinical signs of active vitiligo. The vitiligo extent score (VES) was used to quantify the disease severity of vitiligo (19). We classified disease severity to VES score of < 5 as the disease with less extent of involvement, and VES score of > 5 indicating more advanced disease extent.

Patients in the active stage received dexamethasone 4mg on two consecutive days per week for three months. Topical tacrolimus 0.1% ointment was applied twice per day on all lesions. Patients received low-dose narrowband UVB phototherapy (mean 402 ± 89mj/cm (2) twice per week during follow-up periods. Patient with good response to treatment was defined as those with > 10% of repigmentation with no signs of progression after treatment based on well-documented photograph record. Poor responders are defined as those with < 10% repigmentation and/or progression after treatment. Ten percent (10%) repigmentation was chosen based on previous literature reports (20) and to maximize the statistical power by separating patients’ responses into two groups with similar sizes.

### Suction Blister Interstitial Fluid and Plasma Analytes Collection

Suction blisters interstitial fluid (1cm in diameter, five blisters each chamber) obtained from the lesion and non-lesion skin using the Negative Pressure Instrument Model NP-4 (Electronic Diversities, Finksburg MD). The suction chambers were attached to the skin with a strap. The chambers generate a constant temperature of 40°C with 10-15mmHg negative pressure, and the blisters formed around 60 minutes procedure. Blister fluid with no hemorrhagic content was aspirated using a 1mL insulin syringe through the roof. Approximately 200–800 µl blister fluid could be collected from every individual. The interstitial fluid was collected and stored at -80°C and later sent for a cytokine panel study. For active vitiligo patients, blister interstitial fluid was collected from the new lesions with confetti-like depigmentation and/or trichrome lesions. The blister samples were collected from the perilesional border for stable vitiligo patients, overlapping normal and depigmented skin. Non-lesion blister sampling was performed at the normal-appearing area, confirmed by wood’s lamp with a 10cm distance from the closest depigmented lesion. A 10cc whole blood was collected from vitiligo patients and healthy controls and processed plasma based on standard protocol (21). The plasma was aliquoted into cryovials and stored at -80°C until analysis (22).

### Multiplex Cytokine Analysis With High-Sensitive Meso-Scale Discovery (MSD) and Ultra-Sensitive Single-Molecule Counting Technology (SMC) Immunoassay

We utilized the high-sensitivity Meso Scale Discovery (MSD) electrochemiluminescence assay for the cytokine panel study to measure IFN-γ, CXCL9, CXCL10, CXCL11, CXCL12, IL-1β, IL-13, IL-15, IL-17A, IL-18 for blister fluid analysis and plasma analysis. MSD plates were analyzed on the MS2400 imager (23). The sample volume requirement for MSD is much less than conventional ELISA, enabling more cytokine to be studied in a single blister interstitial fluid analyte (23). The ultra-sensitive single-molecule counting technology (SMC) was used to analyze plasma IFN-γ that are normally lower expressed in the plasma (24). These technology platforms are more sensitive compared to conventional ELISA (Detection range SMC:0.1-10,000pg/mL; MSD:1-10,000pg/mL; high sensitivity ELISA:1-10pg/ml; conventional ELISA:10-1000pg/mL) (24). Both MSD and SMC assays were performed based on the manufacturer’s instructions. All standards and samples were measured in a triplicate manner.

### Statistical Analysis

Continuous variables are expressed as the mean ( ± standard deviation) or median (quartiles) and as count (percentage) for discrete variables. Paired t-test was used to analyze paired lesion, non-lesion skin, and plasma quantitative values. Welch’s t-test or unequal variances t-test is used to test the means of quantitative values for two groups. ANOVA was used for comparison in more than two groups. Pearson correlation analysis explored the linear relationship between two continuous variables for quantitative values. Receiver Operating Characteristic (ROC) curve analysis was performed to determine the sensitivity and specificity of tests performed on plasma and blister samples in active and stable vitiligo. The sample size justification for vitiligo patient and healthy control achieved 74% power to detect the difference between null hypothesis (IFN-γ expression in plasma analyte of vitiligo patients, mean ± SD: 3.56 ± 6.11) and alternative hypothesis (IFN-γ expression in plasma analyte healthy controls mean ± SD: 1.26 ± 0.78) with a significance level of 0.05 using a two-sided two-sample t-test (25, 26).

A power of > 70% is considered sufficient for the study. Statistical analysis was performed with SPSS version 24.0 software for Windows (IBM, Armonk, NY, USA) and GraphPad Prism software. P < 0.05 was regarded as statistically significant.

## Results

### Demographics of Vitiligo Patients and Healthy Controls

We prospectively collected 86 patients with non-segmental vitiligo and 34 healthy controls from the outpatient vitiligo clinic from a tertiary medical center in Taiwan. All patients were of Asian ethnicity. We first obtained plasma and paired blister interstitial fluid from the lesion and non-lesion skin from 33 patients with vitiligo and 12 healthy controls as the defining group and were sent for multiplex cytokine analysis **(** **Table 1****).** Twenty-six patients were in active vitiligo disease activity, and seven were stable. The average age for active vitiligo was 46.30 ± 15.54 years, stable vitiligo 41.57 ± 10.95 years, and healthy controls 40.82 ± 7.43 years. The average disease duration was 8.46 ± 6.77years in active vitiligo and 8.28 ± 6.26 years in stable vitiligo. Subsequently, we further analyzed the plasma from 53 vitiligo patients and 22 healthy controls as the validation group. Among all, 40 patients were in active disease activity, and 13 were stable vitiligo. The mean age of active vitiligo was 45.93 ± 12.12 years, stable vitiligo 42.07 ± 14.08 years, healthy 41 ± 7.23 years. The disease duration was 7.28 ± 6.03 years and 7.92 ± 8.71 years for active and stable vitiligo, respectively.

**Table 1:** Demographics of non-segmental vitiligo patients and healthy controls.

	Paired Blister Interstitial Fluid and Plasma			*p-value*	Plasma			*p-value*
	Active Vitiligo	Stable Vitiligo	Healthy		Active Vitiligo	Stable Vitiligo	Healthy	
Sample size, n	26	7	12		40	13	22	
Female, No (%)	12 (46%)	3 (43%)	5 (41%)	0.82	16 (40%)	5 (38%)	9 (41%)	0.73
Age, (mean (SD), year)	46.30 (15.54)	41.57 (10.95)	40.82 (7.43)	0.29	45.93 (12.12)	42.07 (14.08)	41 (7.23)	0.21
Age at disease onset (mean (SD), year)	37.85 (19.04)	33.27 (12.4)	–	0.55	38.65 (13.87)	34.15 (17.11)	–	0.34
Disease duration (mean (SD), year)	8.46 (6.77)	8.28 (6.26)	–	0.95	7.28 (6.03)	7.92 (8.71)	–	0.76

### Cytokine Expression in Skin Interstitial Fluid of Lesion and Non-Lesion Area and Paired Plasma in Vitiligo Patient Compared to Healthy Control

**Figure 1:**
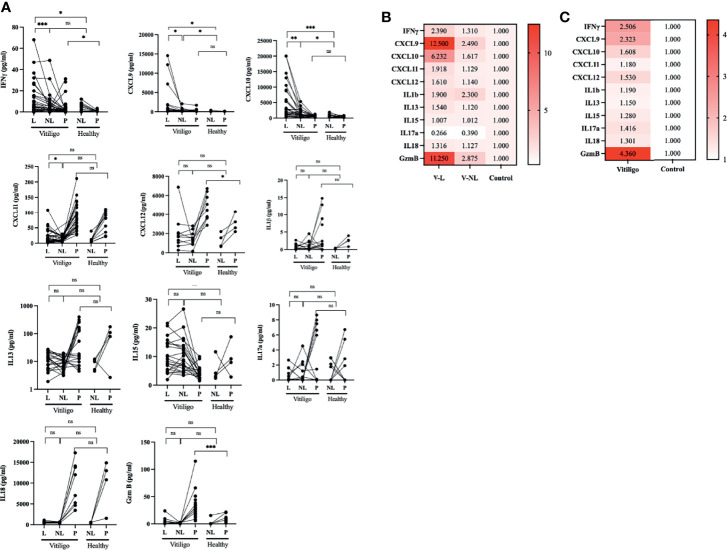
IFN-γ, CXCL9, CXCL10, and GzmB are highly expressed in the skin lesion, and plasma of vitiligo patients compare to healthy controls. **(A)** Cytokine panel expression of the paired lesion and non-lesion skin blister interstitial fluid and plasma analyte in vitiligo patients and healthy controls. **(B)** Relative fold change cytokine panel in skin blisters interstitial fluid analyte in the lesion, and non-lesion skin of vitiligo patients compared to healthy controls. **(C)** Relative fold change of cytokine panel in plasma analyte of vitiligo patients compared to healthy controls. (V, Vitiligo; L, lesion; NL, non-lesion; *p < 0.05, **p < 0.01, ***p < 0.001; ns, non-significant).

Paired blister interstitial fluid from lesion and non-lesion skin areas and plasma was collected from 33 vitiligo patients, consisting of 26 active and seven stable status (**Table 1**). These samples were then analyzed alongside non-lesion skin and plasma samples from ten healthy sex and age-matched volunteers using a selected panel of cytokines with MSD immunoassays. IFN-γ levels proved to be significantly upregulated in lesion skin compare to non-lesion and healthy (vitiligo lesion(V-L):11.66 ± 15.83pg/ml; vitiligo non-lesion(V-NL):6.43 ± 10.04pg/ml; healthy controls(Ctrl): 4.87 ± 3.86pg/ml; *p* = 0.013) and plasma (vitiligo(V): 3.56 ± 6.11pg/ml; Ctrl: 1.48 ± 1.027pg/ml, *p* = 0.023) of vitiligo patients. The expression level is significantly different between lesion skin and non-lesion skin of vitiligo patients (*p* < 0.008) and lesion skin of vitiligo compared to healthy skin (*p* = 0.03), but not between non-lesion skin of vitiligo patients compared to healthy skin (*p* = 0.5) **(** **Figure 1A****).**

IFN-γ signature chemokines, CXCL9 and CXCL10, are highly expressed in both lesion skin and non-lesion skin of vitiligo patients compared to healthy control with statistical significance. (CXCL9: V-L: 461 ± 3386pg/ml, V-NL: 290.2 ± 377.1pg/ml; Ctrl: 162.5 ± 179.5 pg/ml, *p* < 0.05; CXCL10: V-L: 4434 ± 5596 pg/ml; V-NL: 1394 ± 1175 pg/ml; Ctrl: 788.5 ± 493.7 pg/ml, *p* < 0.05). Although exhibiting trends, the plasma level of CXCL9 and CXCL10 are non-significant between both groups. CXCL11 is elevated in the lesion compared to non-lesion skin of vitiligo (V-L:21.83 ± 22.65pg/ml; V-NL: 12.85 ± 7.763 pg/ml, *p* = 0.027). CXCL12 is elevated in the plasma of vitiligo patients compared to healthy controls (V-P:4707 ± 1378 pg/ml; Ctrl-P: 1274 ± 740.5 pg/ml, *p* = 0.03).

Intriguingly, granzyme B (GzmB), a key effector of cytotoxic T cells, is highly expressed in the lesion and non-lesion skin and plasma of vitiligo patients compared to healthy controls. (V-L: 5.17 ± 7.1 pg/ml; V-NL:1.33 ± 0.98 pg/ml; V-P: 27.86 ± 19.55 pg/ml; Ctrl: 10.15 ± 6.61 pg/ml, *p* < 0.001).

By way of comparison, no significant changes in IL-1β, IL-13, IL-15, IL-17A, and IL-18 were observed.

We further analyzed the relative fold change of cytokine expression in vitiligo patients compared to healthy control. **(** **Figures 1B, C****)**. The results show that IFN-γ, CXCL9, CXCL10, and GzmB are highly expressed in the skin blister interstitial fluid and plasma of vitiligo compared to healthy controls.

### Cytokine Expression Profile of Skin Interstitial Fluid and Plasma Samples Are Correlated

**Figure 2:**
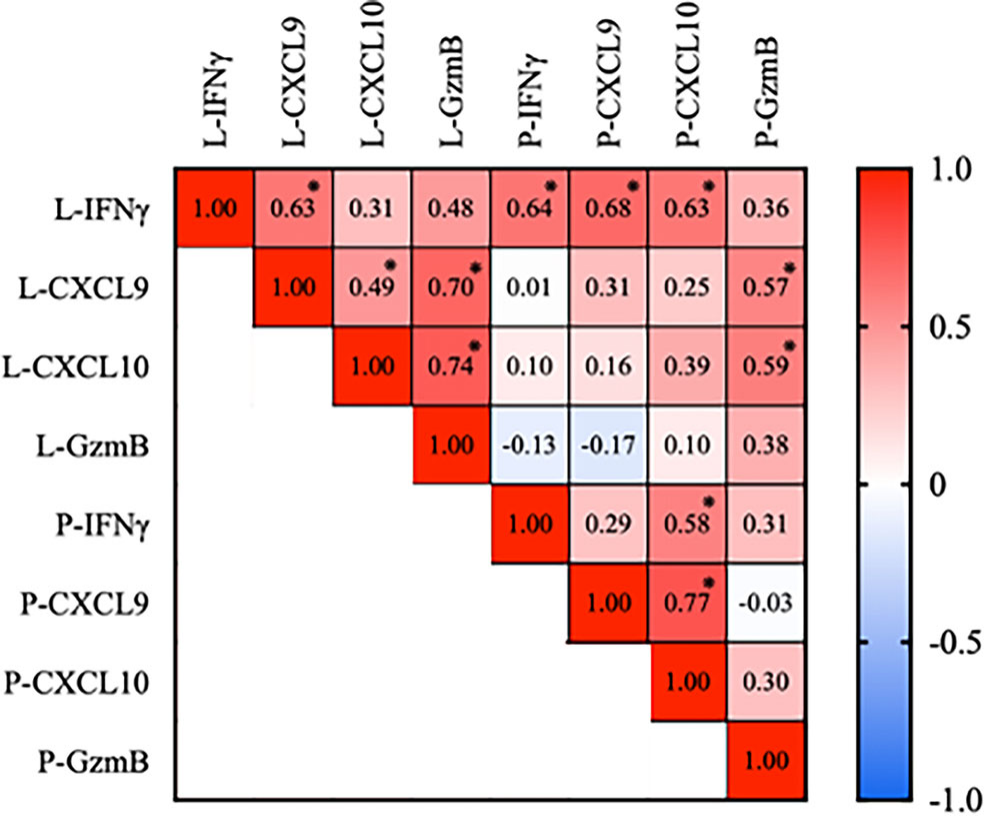
Pearson correlation analysis of IFN-γ, CXCL9, CXCL10, and GzmB expression in skin blister interstitial fluid and plasma analyte in vitiligo patients. Plasma IFN-γ, CXCL9, and CXCL10 expression positively correlated with the skin lesion IFN-γ expression. Plasma Granzyme B is correlated with skin lesion GzmB, CXCL9, and CXCL10. (*p < 0.05, L, lesion; NL, non-lesion; P, plasma).

Subsequently, we performed a correlation analysis of lesion skin and plasma cytokine expression in IFN-γ, CXCL9, CXCL10, and GzmB. There is a strong positive correlation between the expression of IFN-γ in the lesion skin and plasma IFN-γ, plasma CXCL9, and CXCL10 (Pearson correlation coefficient (r); P-IFN-γ = 0.6405, *p* = 0.003; P-CXCL9 r = 0.68, *p* < 0.001; P-CXCL10 r = 0.63, *p* = 0.001). Indicating that plasma IFN-γ, CXCL9 and CXCL10 could be used as markers for lesion IFN-γ. Interestingly, there is a strong correlation between the lesion and plasma GzmB with lesions CXCL9 and CXCL10, but not plasma CXCL9 and CXCL10. The plasma IFN-γ is significantly correlated with plasma CXCL-10, and the plasma CXCL9 is strongly associated with CXCL10 (**Figure 2**)

### IFN-γ, CXCL10, and Granzyme B Are Associated With Disease Activity, and CXCL9 Is Associated With Disease Severity

**Figure 3:**
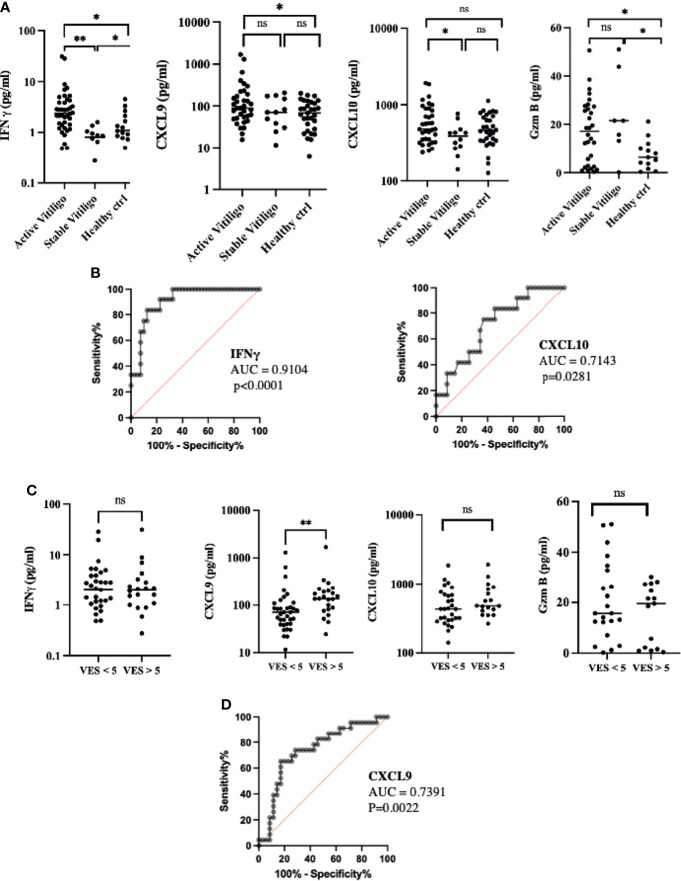
IFN-γ and CXCL10 are correlated with disease activity, whereas CXCL9 is associated with disease severity in vitiligo patients. **(A)** Disease activity: IFN-γ, CXCL9, CXCL10, and GzmB in active and stable vitiligo and healthy control. **(B)** ROC curve for disease activity in IFN-γ and CXCL10, negative data of CXCL9 and GzmB not shown. **(C)** Disease severity: IFN-γ, CXCL9, CXCL10, and GzmB in VES < 5 and VES > 5 vitiligo patients. **(D)** ROC curve for disease severity in CXCL9, negative data of IFN-γ, CXCL10 and GzmB not shown (*p < 0.05, **p < 0.01). Ns, non-significant.

We further study the plasma expression of IFN-γ, CXCL9, CXCL10, and GzmB in active and stable vitiligo compared to healthy control. Plasma IFN-γ is significantly elevated in active vitiligo compared to stable vitiligo and healthy controls. CXCL10 is elevated substantially with active vitiligo compared to stable vitiligo, whereas CXCL9 of active vitiligo is significantly elevated compared to healthy controls but not stable vitiligo. (**Figure 3A**). ROC curve analysis for IFN-γ in active versus stable vitiligo is very significant. (AUC: 0.9104, *p* < 0.0001). In comparison, the AUC for CXCL10 in active versus stable vitiligo is lower than that of IFN-γ (AUC: 0.7143, *p* = 0.0281). (**Figure 3B**) The expression of plasma CXCL9 is elevated considerably with patients with a higher VES score, indicating a more advanced disease severity. IFN-γ, CXCL10, and Granzyme B were not significantly different in both groups. (**Figure 3C**) ROC curve analysis of CXCL9 with disease severity in vitiligo is significant (AUC = 0.739, *p* = 0.0022). (**Figure 3D**)

### Expression Level of IFN-γ Is Correlated With Disease Prognosis

**Figure 4:**
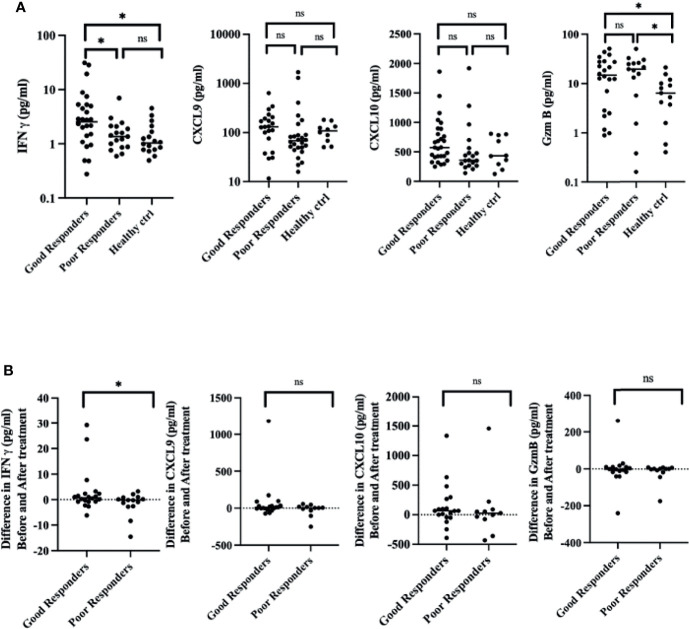
A decrease in IFN-γ expression level before and six months after treatment correlates with good treatment response. **(A)** Cytokine expression of IFN-γ, CXCL9, CXCL10, and GzmB at the initial visit. **(B)** Difference in IFN-γ, CXCL9, CXCL10, and GzmB expression before and six months after treatment. (*p < 0.05). Ns, non-significant.

We investigate the feasibility of IFN-γ, CXCL9, CXCL10, and GzmB as prognosis predictors for vitiligo. The expression level of IFN-γ at the initial visit of good responders (G) is significantly higher than that of poor responders (P), indicating that active disease status has a better response to immunosuppressive treatment (G: 5.22 ± 7.94 pg/ml; P:1.67 ± 1.48 pg/ml; *p* = 0.279) (**Figure 4A**). Good and poor responders had higher GzmB on initial presentation than healthy control. Analysis of sequential cytokine expression at initial visits and six months after treatment reveals that good responders have higher and positive differences of IFN-γ (G: 3.00 ± 7.94 pg/ml, P: -1.67 ± 4.6 pg/ml, *p* = 0.04), whereas CXCL9, CXCL10, and GzmB were non-significant. Indicating that decrease in IFN-γ expression level is positively correlated with the response to treatment (**Figure 4B**).

## Discussion

Vitiligo is an acquired autoimmune depigmentation skin disorder characterized by an unpredictable and refractory course. In current practice, disease activity is mainly assessed by the medical history based on patients’ recall. Clinical markers of trichome sign, confetti-like depigmentation, and type 2b Koebner phenomenon are associated with vitiligo progression (16, 27). However, an individual patient often has various disease activity stages lesions in the clinical realm. Moreover, studies have shown that relapse is a common issue in patients with vitiligo. It has been estimated that 40% of the patients experienced relapse within one year after stopping treatment (28, 29). Hence, a biomarker exhibiting a net effect of disease activity is more practical in evaluating the patient and follow-ups.

In this study, we prospectively recruited an extensive number of vitiligo patients and healthy control to identify the optimal biomarker for vitiligo and as potential guidance for clinical practice and future targeted therapy development. The plasma cytokine profile resembles the whole body inflammation and may not resemble the local tissue inflammation of the skin (6). Hence, we first utilized the suction blister interstitial fluid method to analyze the local tissue inflammation of vitiligo lesion, and non-lesion skin compared to healthy controls. The suction blister interstitial fluid is a less invasive method with analytes derived from interstitial fluid, where many essential biomarkers are expected to be found (30). The method has been utilized to study inflammatory mediators, proteomic expression, drug detection, and biomarkers in local skin diseases. Suction blister fluid has been used as a source of biomarker study in epidermal necrolysis (31) and autoimmune blister diseases (32). However, the study of suction blister fluid in vitiligo is limited (6, 8). Moreover, there is a lack of extensive multiplex cytokine panel analysis in vitiligo lesion, non-lesion skin compared to healthy control due to the limited amount of obtained blister interstitial fluid analyte.

Our study showed that IFN-γ and its’ signature cytokines, including CXCL9 and CXCL10, and GzmB, are most highly expressed in the vitiligo patients’ blister and plasma compared to healthy control. By way of comparison, no significant changes in IL-1β, IL-13, IL-15, IL-17A, IL-18 were observed. ROC curve analysis shows that the levels of IFN-γ appear to be most sensitive and specific in diagnosing active vitiligo, followed by CXCL-10 and GzmB. CXCL-9 was sensitive and specific in diagnosing vitiligo disease severity. A decrease in the expression level of IFN-γ is positively correlated with treatment response.

IFN-γ and its’ signature chemokines CXCL9 CXCL10 are critical in the pathogenesis of vitiligo for recruiting autoreactive cytotoxic T lymphocytes (CTLs) (1, 33). Studies found that CTLs secrete IFN-γ that induced keratinocyte secretion of CXCL-9 and CXCL10 which orchestrates the migration of T cells to the vitiligo lesion. IFN-γ also participates in direct inhibition of melanogenesis in a leprosy model and contribute to increased oxidative stress and melanocyte cell death in primary normal melanocytes (34, 35). Many studies have shown that CXCL9 and CXCL10 are elevated in the suction blisters interstitial fluid and plasma analytes and act as potential biomarkers for differentiating between active and stable vitiligo (9, 36). By contrast, due to the detection limit with conventional ELISA, the study of IFN-γ as a biomarker is scarce (37). Detail studies of the association of these cytokine expressions with vitiligo clinical activity and prognosis are also limited. In this study, we utilized ultra-sensitive immunoassay- MSD and SMC, with low detection limits that are more suitable for samples with low endogenous levels of cytokines (23, 38). Our results show that IFN-γ is the most sensitive and specific marker in predicting disease activity and prognosis, supporting the critical role of IFN-γ in the pathogenesis of vitiligo and targeting therapy directed against IFN-γ is essential to halt disease progression.

In addition, this study also uncovered the significantly elevated expression profile of GzmB in the skin and plasma is less reported in vitiligo pathogenesis. GzmB, a serine protease secreted by CTLs and natural killer cells, exhibits a pathological role in many autoimmune skin disorders, particularly in skin blisters diseases (39, 40). GzmB polymorphism has been reported in the previous literature in non-segmental vitiligo (41, 42) (43). Furthermore, aside from pro-apoptotic function, studies have shown that GzmB is responsible for the cleavage of the extracellular matrix that contributes to the collapse of hair follicle immune privilege in alopecia areata, a close disease to vitiligo (44). Further studies are needed to investigate the pathological role of IFN-γ and GzmB in the pathogenesis of vitiligo, aside from merely a marker for cytotoxic T cells.

The current study has several limitations. First, this is a single-center study; there was a potential selection bias of the patients in the cohort. Secondly, we only selected the most significantly expressed cytokines: IFN-γ, CXCL9, CXCL10, and GzmB for the expanded plasma study in correlation with the disease course. A more extensive sample-sized study may uncover additional biomarkers not detected in the current study. The increased level of IFN-γ in the plasma analytes may be caused by systemic inflammatory disease that is not discovered during the initial screening. Furthermore, we also did not evaluate lesions from glabrous vitiligo skin and those with extensive leukotrichia (>30%) or segmental vitiligo as these subtypes have been shown to respond poorly to medical treatment.

In conclusion, our results show that IFN-γ, CXCL9, CXCL10, and GzmB are highly expressed in vitiligo patients’ lesion skin and plasma and may serve as biomarkers for the clinical activity, severity, and prognosis prediction in vitiligo patients. A combination approach using these four panels and a high-sensitive cytokine array could be implemented in clinical practice to assess vitiligo treatment response and outcome objectively.

## Data Availability Statement

The raw data supporting the conclusions of this article will be made available by the authors, without undue reservation.

## Ethics Statement

The studies involving human participants were reviewed and approved by Chang Gung Memorial Hospital (IRB no: 201802265A3). The patients/participants provided their written informed consent to participate in this study.

## Author Contributions

CN: Patients enrolling, demographical information and clinical parameters, project development and therapeutic response data, statistical analyses, drafting and revision of the manuscript, and funding. Y-CC: Clinical parameters, blister collecting, and immunoassay. Y-PC: Immunoassay. P-HC: Bioinformatics and statistical analyses and immunoassay. W-HC: Conceptual development of the project, revision of the manuscript. C-LK: Conceptual development of the project. Revision of the manuscript and funding. All authors contributed to the article and approved the submitted version.

## Funding

This study was supported by the Chang Gung Memorial Hospital Research Funding Program (CMRPGBJ0023) and the Ministry of Science and Technology Taiwan (MOST 110-2314-B-182A-155-MY3).

## Conflict of Interest

Author P-HC is/was employed by Elixiron Immunotherapeutics Inc. C-LK is/was the advisory board member of Elixiron Immunotherapeutics Inc.

The remaining authors declare that the research was conducted in the absence of any commercial or financial relationships that could be construed as a potential conflict of interest.

## Publisher’s Note

All claims expressed in this article are solely those of the authors and do not necessarily represent those of their affiliated organizations, or those of the publisher, the editors and the reviewers. Any product that may be evaluated in this article, or claim that may be made by its manufacturer, is not guaranteed or endorsed by the publisher.
