# Supplementary material for: Skin Interstitial Fluid and Plasma Multiplex Cytokine Analysis Reveals IFN-γ Signatures and Granzyme B as Useful Biomarker for Activity, Severity and Prognosis Assessment in Vitiligo
Source: Front Immunol. 2022 Apr 7;13:872458. doi: 10.3389/fimmu.2022.872458 (PMC9021541; doi:10.3389/fimmu.2022.872458)
Supplement: Supplementary file 1 [file Image_1.pdf]

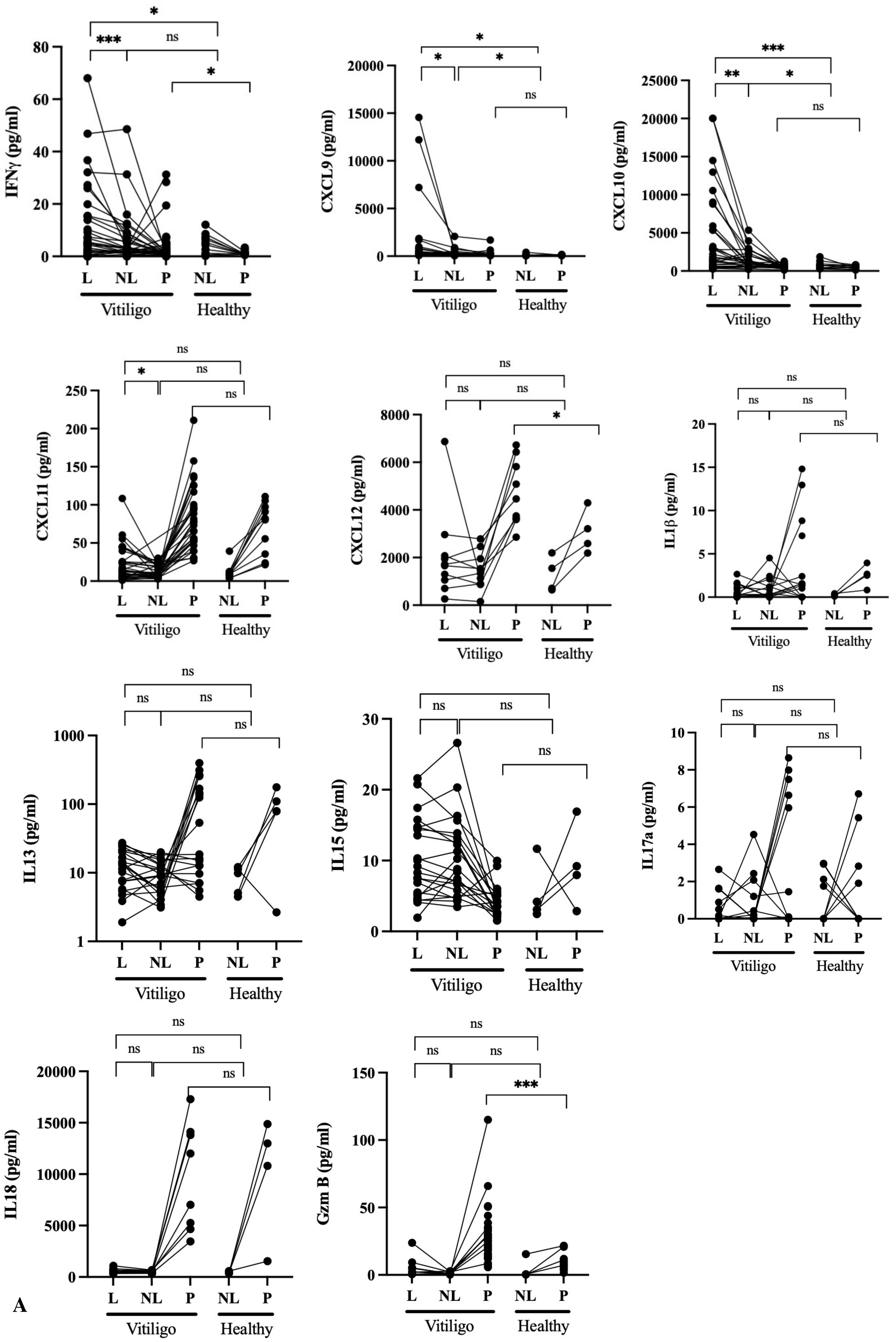
IFNγ (pg/ml)
CXCL9 (pg/ml)
CXCL10 (pg/ml)
CXCL11 (pg/ml)
CXCL12 (pg/ml)
IL1β (pg/ml)
IL13 (pg/ml)
IL15 (pg/ml)
IL17a (pg/ml)
IL18 (pg/ml)
Gzm B (pg/ml)
L
NL
P
Vitiligo
Healthy
A

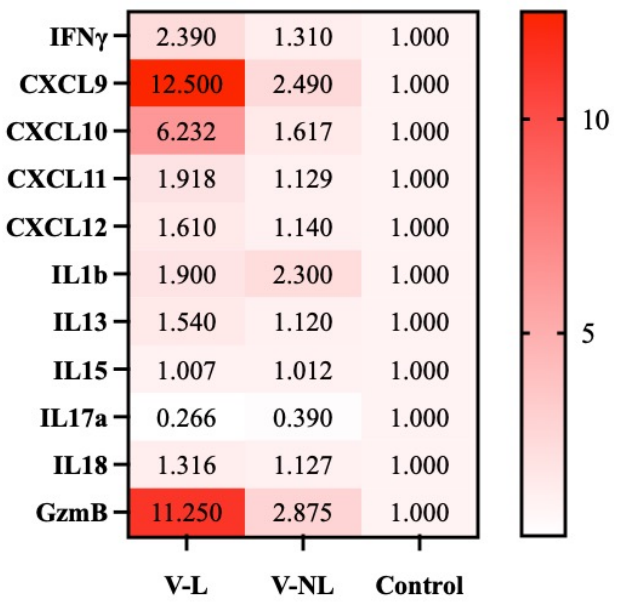

B

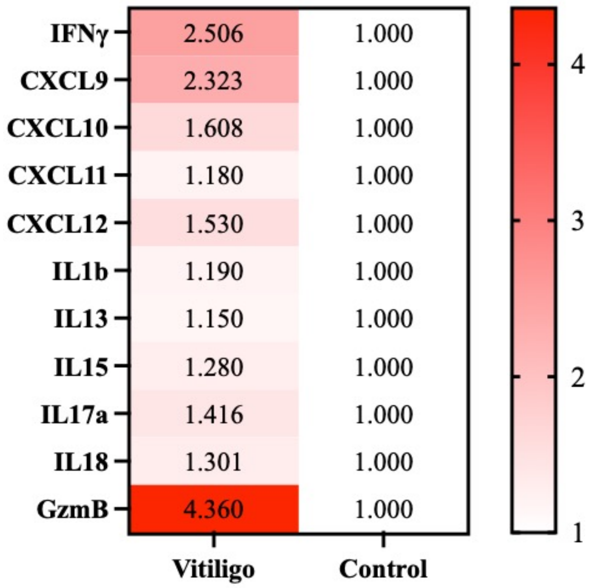

C

Figure 1 IFNγ, CXCL9, CXCL10 and Granzyme B are highly expressed in the skin lesion and plasma of vitiligo patients compare to healthy controls. **A.** Cytokine panel expression of paired lesion and non-lesion skin blister interstitial fluid and plasma analyte in vitiligo patients and healthy controls. **B.** Relative fold change cytokine panel in skin blisters interstitial fluid analyte in lesion and non-lesion skin of vitiligo patients compare to healthy controls. **C.** Relative fold change of cytokine panel in plasma analyte of vitiligo patients compare to healthy controls. (V:Vitiligo, L: lesion, NL: non-lesion; * p<0.05, ** p<0.01,*** p<0.001; ns: non-significant)

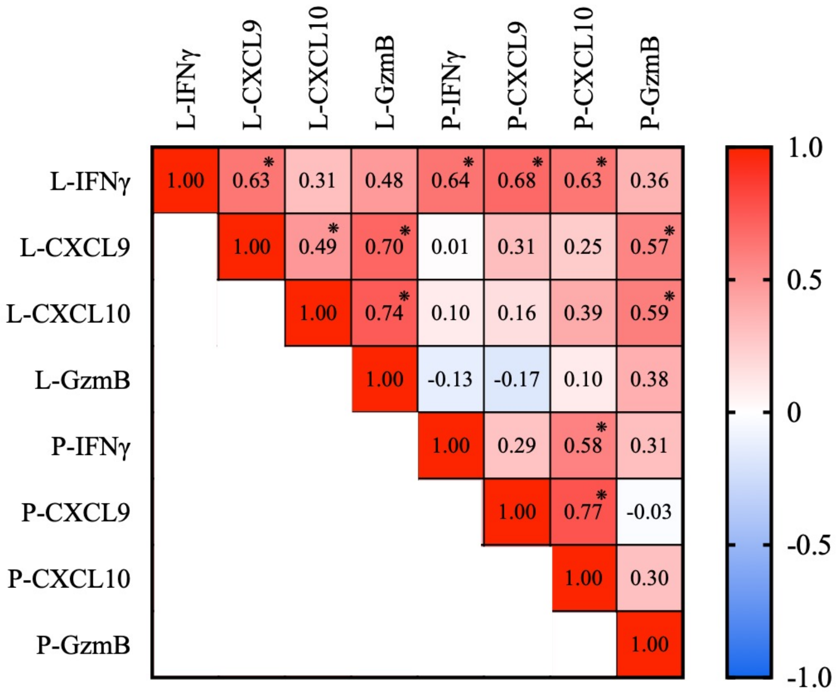

Figure 2. Pearson correlation analysis of IFNγ, CXCL9, CXCL10 and GzmB expression in skin blister interstitial fluid and plasma analyte in vitiligo patients. Plasma IFNγ, CXCL9 and CXCL10 expression is positively correlated with the skin lesion IFNγ expression. Plasma Granzyme B is correlated with skin lesion GzmB, CXCL9 and CXCL10. (* p<0.05, L: lesion, NL:non-lesion, P:plasma)

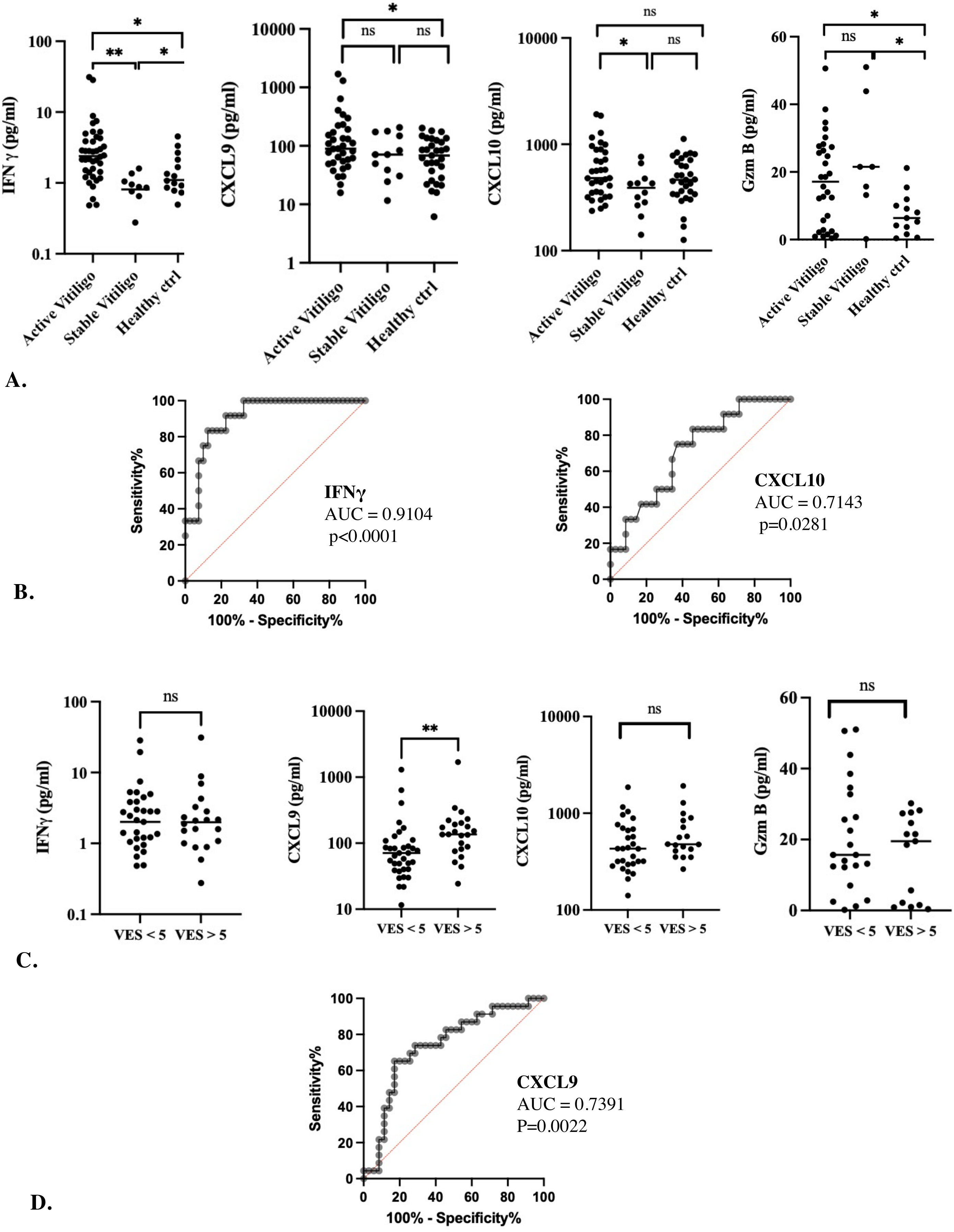

Figure 3. IFNγ and CXCL10 are correlated with disease activity, whereas CXCL9 is associated with disease severity in vitiligo patients. **A.** Disease activity: IFNγ, CXCL9, CXCL10, and GzmB in active and stable vitiligo and healthy control. **B.** ROC curve for disease activity in IFNγ and CXCL10, negative data of CXCL9 and GzmB not shown. **C.** Disease severity: IFNγ, CXCL9, CXCL10, and GzmB in VES <5 and VES >5 vitiligo patients. **D.** ROC curve for disease severity in CXCL9, negative data of IFNγ, CXCL10 and GzmB not shown (* $p<0.05$, ** $p<0.01$,*** $p<0.001$)

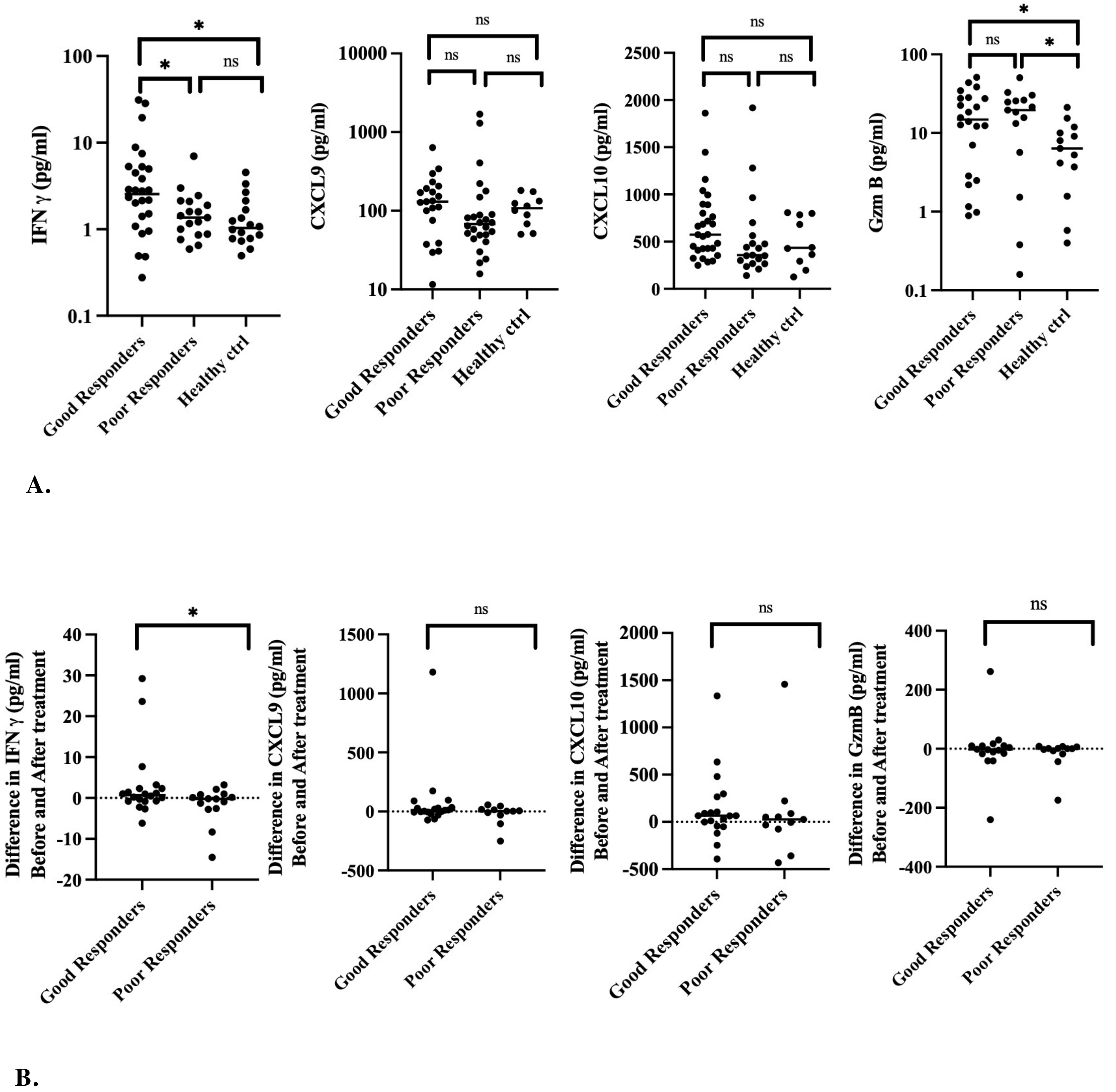

Figure 4. A decrease in IFN-γ expression level before and six months after treatment correlates with good treatment response. **A.** Cytokine expression of IFN-γ, CXCL9, CXCL10, and GzmB at the initial visit. **B.** Difference in IFN-γ, CXCL9, CXCL10, and GzmB expression before and six months after treatment. (* $p<0.05$, ** $p<0.01$,*** $p<0.001$)

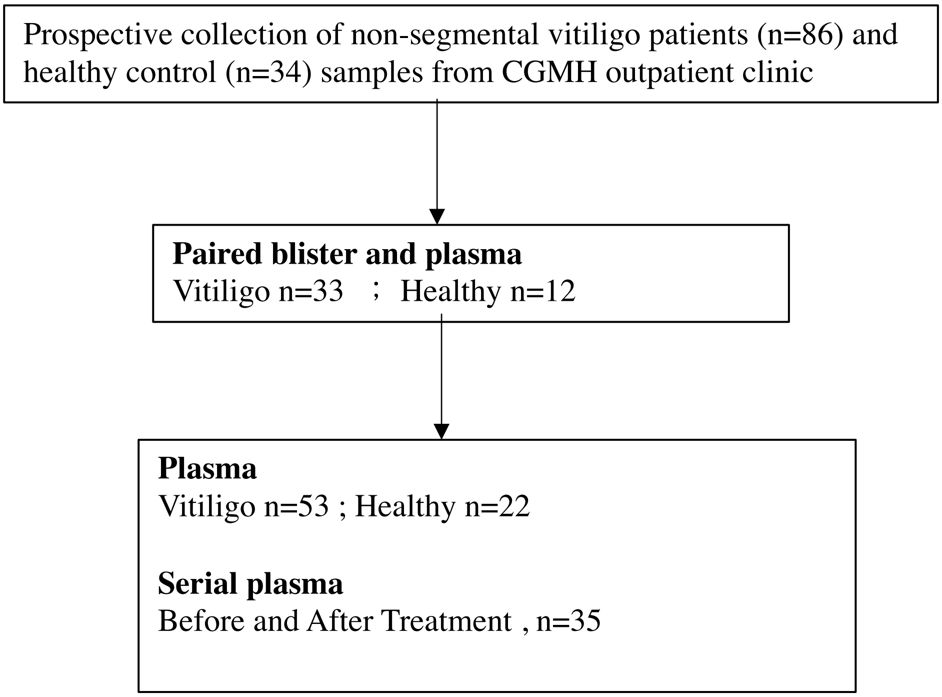

Supplement Fig 1. Patient collection flow chart.
